# False Impressions

**Published:** 1889-01-19

**Authors:** Caroline Fothergill

**Affiliations:** Author of "An Enthusiast," "A Voice in the Wilderness," etc.


### FALSE IMPRESSIONS.

By Caroline Fothergill, Author of "An Enthusiast," "A Voice in the Wilderness," etc.

*(Continued from p. 241.)*

### Chapter XV.—Laura and Edith.

Before Beatrice and Laura paid their return call in Queen Street, the annual meeting of the Murray Society was held. It was rather a brilliant affair, and took the form of a conversazione at the Museum. The work done by the committee was making itself felt, and was creating a good deal of interest in other parts of the country besides Mayfield. There was no lack of help of a thoroughly competent kind, and everyone connected with the Museum was feeling hopeful and encouraged.

Beatrice and Laura were at the meeting. Laura had never been in the old house before, and what she saw there was a revelation to her, but she kept her feelings to herself, and did not appear particularly impressed by anything she saw.

George was there also, accompanied by Edith. The girl was getting used to going out with him now, and yet each occasion brought the same ecstatic feeling, the same thrill of joy at the thought of so much time spent with him, under his protection, the same heart-throbbing of joyous anticipation, the same fulness of content in the reality. It was a dangerous state to be in.

By this time people had begun to notice her, and to wonder who she was. Her whole appearance was too charming for her to remain hidden, and her constant appearance with George Murray, and the attention he paid her—that is, he looked after her, saw that she was amused, and out of the crush, and had refreshments when she wanted them—caused people to speculate about her. George introduced her to very few people, and when he did, called her merely "Miss Lester," without any explanation. People were therefore thrown on their own resources, and the rumour got about that she was Murray's ward, young and wealthy, judging from her dress and general appearance, and probably destined by him for himself. Hence the secluded life she led, and the way in which he guarded her when they were out together.

Laura was one of the first to hear the rumour some time before the evening with which we are at present occupied, and she kept her knowledge to herself, and anticipated a pleasant occupation in watching how things turned out. She did not believe a word of it, having, as we know, her own pet project: but she said nothing of her unbelief, she had long ago learnt the wisdom of letting people talk, and keeping her own counsel; but she was very curious to see Edith, and, above all, to see her with George. It was partly the hope that this wish might be fulfilled which had brought her out this evening.

She had not yet seen them, and was beginning to brace herself up to a disappointment. She was standing talking to Beatrice, and the two women, both so beautiful and in such different ways, had already attracted a good deal of attention, even among people who knew them. Laura was in black, Beatrice in white, which increased the striking and curious contrast between them. Beatrice's eyes were on her companion's face; Laura, turning her restless eyes upon everybody and everything in search of the two people she wanted to see, suddenly caught sight of George coming along with Edith, her slight, girlish figure seen to great advantage in a dress of palest blue. Mrs. Ledward chuckled to herself, and waited to see what would happen.

Nothing very exciting on the surface happened. Beatrice did not see them coming until they were close to, and was going to just bow and draw aside; but George, whose whole face changed at the sight of her, stood still, and held out his hand.

"Good evening, Miss Stanford," he said; "our old house looks completely transformed, does it not?"

"I wish we had been able to let the people in as well," she answered; "it seems inconsistent with our principles to keep it to ourselves, especially on an occasion like this."

"Well, you see," was the reply, "we had to take into consideration the question of space, but I hope —— (naming a distinguished man of letters, who was the guest of the evening) will give them an address another time. What we want to-night is ——" and almost without knowing it they launched into conversation.

Laura watched them with a smile, not wholly free from irony.

"Let yourselves go; yes, let yourselves go," she was saying to herself. "It is a sight for the gods. If you would only show each other your true selves we should hear no more rumours about this little girl."

It gave her only keen pleasure to see how completely she and Edith were forgotten. She was a woman of the world, trained to accept the inevitable with a jest and a shrug of the shoulders; besides, in this case, that which, if it had a fair chance, was likely to prove the inevitable, was her heart's desire. But Edith was not a woman of the world, and casting a glance at her as she spoke the last words in her mind, Mrs. Ledward almost laughed aloud as she saw the look—puzzled, resentful, and yet pitiful—on Edith's face. She turned to her at once, and with a spice of cruelty in both words and intention, said:

"You see how completely we are forgotten. Under the circumstances I think we may venture to make friends without the preliminary ceremony of an introduction. Come and sit by me, my dear, and let us talk."

There was malice, raillery, and kindness all together in her tone, and withal a certain authority which Edith was utterly powerless to resist. Feeling a little unhappy, she yet seated herself by Mrs. Ledward's side and made no attempt to baffle that little lady's curiosity.

"What is your name, my dear?" she began, looking at Edith over her fan with her bright, laughing eyes. "As we are English, we must know one another's names or we shall not be able to talk comfortably. My name is Mrs. Ledward; what is yours?"

"Edith Lester," answered the girl, colouring a little under the fixed gaze of her companion.

"Edith," repeated Mrs. Ledward, "you have the look of an Edith. Have you ever observed that girls called Edith almost always have a good deal of trouble and disappointment in their lives?"

"I have been told that everyone has," was Edith's answer, given with a simplicity which amounted to cleverness.

Mrs. Ledward suppressed a laugh. The answer had both pleased and amused her, and she went on with her catechism.

"I have seen you before with Mr. Murray. Do you live at his house? Are you a relative of his?"

Edith had a dim feeling that these questions were scarcely fair, but she did not know how to avoid answering them, so she replied truthfully:

"I live with Mr. Murray's sister; she is my aunt."

"Oh, then he is your uncle," said Mrs. Ledward promptly.

"No, he is not related to me at all. His sister married my father's eldest brother."

"I see," said Mrs. Ledward drily, "related, and yet not related, just as you please. Very convenient, I am sure."

Edith coloured and said nothing, and Laura sat thinking. She had seen Edith's expression when George first spoke to Beatrice, and she noticed the quick way in which she repudiated the idea of there being any relationship between them. Perhaps Edith had heard the rumour. If so, her foolish young hopes must be nipped in the bud. She hardened her heart, and said:

"I suppose you are very fond of him? he seems very kind, and treats you just like a daughter."

She meant well; her intention was to give Edith a hint, but Edith refused to take it. She coloured again and half turned away from her companion, saying:

"He is very kind, but I don't think he treats me like his daughter; he is not much older than I am."

Mrs. Ledward made a grimace behind her fan, and said within herself:

"Worse and worse; she must be put down."

But before she could speak, Beatrice and George having apparently finished their lengthy conversation, returned to themselves and looked round for their forgotten companions. Laura could have laughed aloud at the sight of Beatrice's absent expression, as she said:

"Oh, there you are, Laura! I am so glad. I was afraid you had left me, and it is time we went."

Neither did George look much more in full possession of his senses as he turned to Edith, saying mechanically:

"Come, Edith, and I will find your wrap. It is getting late; you look tired."

The habit of obedience was so strong in Edith that, she put her hand upon George's arm and let him lead her away. She was glad it was time to go, the evening contained no further enjoyment for her. She was a little bewildered at what had happened—their sudden meeting with the two ladies whom she had at once recognised as the same who had so impressed her at the flower show, her companion's abrupt halt and address to the one whom she knew he would call Miss Stanford, the way in which the two had become instantly absorbed in conversation, to the total neglect of everyone and everything else, and her strange interview with the little keen-tongued lady, who had told her her name was Mrs. Ledward. All these things had rather upset Edith's equanamity. She had on these occasions been for so long the one especial object of George's care that she could scarcely realise that she had been left all at once to take care of herself, and apparently forgotten into the bargain, for she had seen the absent way in which George had looked at her when he had finished talking to Miss Stanford. She knew perfectly well that he had scarcely seen her. Both were silent during the drive home. George asked none of the questions he usually did as to whether she had enjoyed herself, what had interested her most, and so on. He leaned back in his corner, silent and absorbed, and she did the same, being oppressed with a ridiculous feeling, as if she must cry, and all the time she kept hearing Mrs. Ledward's mocking words, "You see, we are completely forgotten." She had never in her life felt so unhappy, and she longed to be at home again and alone in her own room. Nor when George did open his lips were the words he said calculated to restore her peace of mind. He was silent until the carriage stopped at the door, and then he roused up and looked at her with the smile she knew so well, but now seemed to cut her to the heart, saying:

"Did you enjoy yourself, Edith? I think this is the best evening we have had."

He did not even notice that she gave him no answer.

Laura laughed to herself as Beatrice hurried her away. She said nothing then, but it was still early when they reached home, and as often happened, they sat late talking over the evening they had just spent.

"Mr. Murray has courage," began Laura. "He has risen in my estimation, I admire his valour. If anyone ever looked like an utterly unapproachable human iceberg, it was you when you bowed to him this evening; yet he spoke."

Beatrice said nothing. Her cheeks were a little flushed, her eyes brilliant; she looked radiantly beautiful.

"You might have introduced him to me," went on Mrs. Ledward. "I should like to make his acquaintance."

"I beg your pardon," said Beatrice gravely. "I did not think of it."

Laura smiled. "That was evident," she said; "so evident that I should never think of bearing you any malice. I can wait; no doubt another opportunity will occur."

"Perhaps!" was Beatrice's only moderately encouraging answer.

"I do not see now how you can avoid giving him an invitation to your afternoons."

"I shall not give him an invitation."

"Did you see the girl with him to-night?"

"Yes; I saw her."

"What did you think of her?"

"She is very pretty, and very young, I should think."

"You have exactly described her; and these young girls are often very tiresome. I talked to her and got a good deal of information from her."

"I am sure you did," said Beatrice, blandly.

"I know you think I am inquisitive; but I do not mind."

"I think," said Beatrice, gathering together her gloves and fan before leaving the room, "that you talk and think a great deal too much about Mr. Murray. It is quite unnecessary, as far as I am concerned; he is by no means the object of absorbing interest to me which you seem to believe; and, apart from that, I think it is rather vulgar."

*(To be continued.)*

